# Comparison of Laboratory and Daily-Life Gait Speed Assessment during ON and OFF States in Parkinson’s Disease

**DOI:** 10.3390/s21123974

**Published:** 2021-06-09

**Authors:** Marta Francisca Corrà, Arash Atrsaei, Ana Sardoreira, Clint Hansen, Kamiar Aminian, Manuel Correia, Nuno Vila-Chã, Walter Maetzler, Luís Maia

**Affiliations:** 1 Abel Salazar Biomedical Sciences Institute (ICBAS), University of Porto, 4050-313 Porto, Portugal; mcorreia.neurologia@chporto.min-saude.pt (M.C.); luismaia.neurologia@chporto.min-saude.pt (L.M.); 2 University Hospital Santo Antonio of Porto (CHUP), 4099-001 Porto, Portugal; ana.sardoeira@gmail.com (A.S.); nunovilacha@hotmail.com (N.V.-C.); 3 Laboratory of Movement Analysis and Measurement, Swiss Federal Institute of Technology in Lausanne (EPFL), 1015 Lausanne, Switzerland; atrsaei.arash@epfl.ch (A.A.); kamiar.aminian@epfl.ch (K.A.); 4 Department of Neurology, Christian-Albrechts-University, 24118 Kiel, Germany; c.hansen@neurologie.uni-kiel.de (C.H.); w.maetzler@neurologie.uni-kiel.de (W.M.); 5 Institute for Research and Innovation in Health (i3s), University of Porto, 4200-135 Porto, Portugal

**Keywords:** remote patient monitoring, medication states, Parkinson’s disease, lab vs. home, wearable sensors, human gait, gait speed

## Abstract

Accurate assessment of Parkinson’s disease (PD) ON and OFF states in the usual environment is essential for tailoring optimal treatments. Wearables facilitate measurements of gait in novel and unsupervised environments; however, differences between unsupervised and in-laboratory measures have been reported in PD. We aimed to investigate whether unsupervised gait speed discriminates medication states and which supervised tests most accurately represent home performance. In-lab gait speeds from different gait tasks were compared to home speeds of 27 PD patients at ON and OFF states using inertial sensors. Daily gait speed distribution was expressed in percentiles and walking bout (WB) length. Gait speeds differentiated ON and OFF states in the lab and the home. When comparing lab with home performance, ON assessments in the lab showed moderate-to-high correlations with faster gait speeds in unsupervised environment (*r* = 0.69; *p* < 0.001), associated with long WB. OFF gait assessments in the lab showed moderate correlation values with slow gait speeds during OFF state at home (*r* = 0.56; *p* = 0.004), associated with short WB. In-lab and daily assessments of gait speed with wearables capture additional integrative aspects of PD, reflecting different aspects of mobility. Unsupervised assessment using wearables adds complementary information to the clinical assessment of motor fluctuations in PD.

## 1. Introduction

Parkinson’s disease (PD) is a chronic and progressive neurodegenerative disorder characterized by impairment of mobility and gait, with severe consequences on quality of life. Motor symptoms and gait impairment are mainly caused by loss of dopaminergic neurons in the substantia nigra, decreasing dopamine levels in the brain [1]. Thus, current treatments of PD focus on increasing dopamine delivery: among the main dopaminergic medications, levodopa is considered the gold standard therapy [2]. Up to 50% of PD patients within two years of levodopa therapy may begin to experience mild motor fluctuations [3]. Motor fluctuations represent alternations of periods of good dopaminergic effect, with adequate control of movements (the perception of this state by the affected patient is called “ON state”) to others of poor control and significant worsening of motor symptoms (comparably, this perception by the affected patient is called “OFF state”) [4].

Reduction of motor fluctuations is an important indicator to evaluate the effectiveness of pharmacological interventions. The most popular tool to quantitatively assess motor fluctuations is the motor part of the Unified Parkinson’s Disease Rating Scale (UPDRS, and the Movement Disorder Society-revised version thereof), a validated clinical rating scale of PD symptoms that includes both historical information and clinical examination for ON and OFF states [5]. However, clinical scores do not necessarily provide representative data on the patient’s daily performance at home. An alternative method of tracking motor fluctuations is to ask patients to fill a diary differentiating various symptoms during the day, and to rate and define their current status of being at ON state or at a decreased medication effect by self-perception. This method has several limitations, including recall bias, reduced compliance, and the need to be accurately compiled to have valid and interpretable data [6]. In addition, these methods do not involve quantitative measures of movements. Monitoring objective gait parameters to track motor fluctuations may compensate these subjective limitations, and allow more sensitivity, accuracy and reproducibility [7].

More recently, wearable health technologies have been developed with the possibility to investigate PD symptoms at a new level of granularity and in novel environments that have previously not been covered by clinical evaluations [8]. In particular, inertial measurement units (IMUs) are electronic devices worn on the body that can detect movements and successfully estimate spatial-temporal parameters, using a combination of accelerometers, gyroscopes and sometimes magnetometers. Thanks to the reduced size and costs of their components, they are easy to wear and low-cost tools for movement analysis [8]. Compared to more complex equipment such as 3D optical motion capture systems, IMUs are less time consuming and do not require specific expertise to use. In addition, complex tools are used only in clinical settings due to their high cost and complexity of technology, and do not often represent the full gait complexity [9]. The use of inertial measurement units (IMUs) indeed makes it now possible to investigate motor features such as gait and motor performance in unsupervised conditions such as the domestic environment. This may enable a passive collection of clinically important information, such as durations of medication ON and OFF states, in natural environments of individuals [10,11,12]. Among the parameters extracted from IMUs, gait speed was shown to be one of the most reliable predictors of mobility [13], risk of falling [14,15], and loss of independence [16], as well as a powerful indicator of changes in performances over time [17]. Gait speed is a critical measure of gait function for different pathologies [13,18,19]. Stratification of gait activity in the home environment in walking bouts (WBs) of different lengths seems to provide additional useful insight into mobility performance [20].

It has been observed that gait speed can significantly differ when analyzed at home or in the laboratory [13,19,21,22,23,24]. Carcreff et al. [13] showed lower gait speed values in daily life compared to lab in a group of children with cerebral palsy. In this study, children were asked to walk barefoot during the lab assessment, which may generate great differences when comparing both assessments. Two other studies [19,23] have compared supervised and unsupervised gait speeds in the elderly. A weak association between daily-life- and lab-obtained gait speed was found by Takayanagi et al. [19], with average daily gait speed being significantly lower than lab speed. De La Camara et al. [23] showed an association with speed and physical, mental and cognitive health outcomes, and highlighted that clinically obtained gait speed can underestimate or overestimate habitual gait speed. All of these studies used IMUs to detect mobility, but they all presented limitations regarding the type of gait tasks used for the lab assessment. For example, distance walked in the lab was between 2.44 and 10 m, which can be considered too short to be compared with daily gait speed. Even in PD, it has been shown that supervised instruments to measure motor symptoms do not strongly reflect daily-living activity [21]. For example, no significant correlation was observed between lab and home gait parameters in a study by Toosizadeh et al. [22], but the small sample size and methodological differences (one single sensor on the sternum) between lab and home assessments of the study may have affected the accuracy of the results. Therefore, it is still unclear whether supervised and unsupervised assessments are as sensitive when used to identify motor fluctuations in PD. Precise information on the degree of association between supervised and unsupervised assessments of motor fluctuations is still lacking. Improving the understanding of gait disabilities with additional information about daily-life performances from IMUs is essential to enhance clinical care, design personalized interventions and overcome limitations concerning questionnaires and self-reported diaries.

For these reasons, in the present study, we compared gait speed from supervised (in the laboratory) and unsupervised (at home and daily-life conditions) assessments to determine the degree of association of the different medication states in PD patients. In particular, we tested whether gait speed in unsupervised environments discriminates ON and OFF states, and investigated which supervised tests most accurately represent home performance during both medication states, also in relation to clinical scores.

## 2. Materials and Methods

### 2.1. Patients

PD patients diagnosed by a movement disorder specialist based on the UK Brain Bank criteria [25] were recruited. The following exclusion criteria were applied:

- Older than 90 years of age;
- Dementia;
- Any relevant gait-impairing health issue other than PD;
- Unable to walk a distance of 20 m;
- Not taking anti-parkinsonian medications in the past month;
- Difference of less than 2 points between ON and OFF in the UPDRS-III, in order to consider the minimum clinically significant difference between states [26,27].

Demographic data and information on medication intake were collected [28]. The main characteristics of the population are presented in the results section. All subjects gave their informed consent for inclusion before they participated in the study. The study was conducted in accordance with the Declaration of Helsinki, and the protocol was approved by the Ethics Committee of Centro Hospitalar Universitário do Porto (CHUP) with identification code 2018.087(076-DEFI/076-CES).

### 2.2. Data Collection

**Figure 1:**
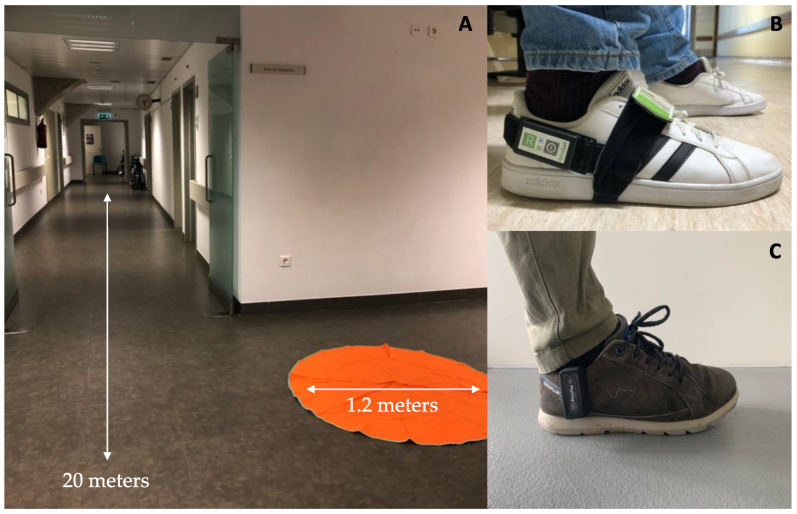
(**A**) Lab setting for the supervised assessment: 20 m walking and circular walking tasks. (**B**). Sensor positioning for lab assessment. (**C**). Sensor positioning for unsupervised assessment.

During the first day of assessment, participants were first evaluated with UPDRS part III (a medical professional evaluates actual motor performance) in their OFF state in the morning, at least 12 h after the last dose of dopaminergic medication intake. They were then equipped with two synchronized RehaGait IMUs (Hasomed GmbH, Magdeburg, Germany), each containing a tri-axial gyroscope and tri-axial accelerometer (Figure 1). These sensors were located on both feet, recording data with a sampling frequency of 100 Hz. Participants were asked to perform the following gait tasks: a 20-m straight walking test at normal pace and at fast pace; and a circular walking task, which is walking at normal pace three times around a circular carpet of 1.2 m diameter, at both left and right directions (Figure 1). Then, participants were asked to take their usual first dose of dopaminergic medication, and the same assessment (UPDRS scores and gait assessment) was performed between 1 and 2 h after intake of medication.

During the second day of assessment, participants wore a Physilog^®^ 5 IMU (Gait Up, Lausanne, Switzerland) on the right foot between 9:00 and 9:30 a.m. (Figure 1). The device comprises a triaxial accelerometer and gyroscope with a sampling frequency of 128 Hz. For the following 12 h (during a weekday), participants were then asked to keep wearing the IMUs and perform usual daily activities in their domestic environment, including their usual outdoor activities. For convenience, the terms ‘home’ or ‘domestic environment’ will include outdoor activities during the day of the assessment.

Both inertial sensors used in the study showed the same technical characteristics and measure the same type of data, guaranteeing no differences in terms of results and set-ups (Figure 1). In addition, participants were asked to fill in a diary over the day to report the times of dopaminergic medication intake. To ensure a precise documentation of time and quantity of dopaminergic medication intake, caregivers were also instructed to monitor the diary record.

### 2.3. Data Processing and Extracted Parameters

Raw data were processed using Matlab R2020b (MathWorks, Nantick, MA, USA). To analyze gait speeds, the raw data of the IMU from the right foot was used for both supervised and unsupervised assessments. To ensure no systematic biases between assessments in terms of heterogeneity, usage and accuracy, raw data of accelerometer and gyroscope from the two assessments were processed with the same algorithm to obtain gait speed [29]. This algorithm had been validated in a previous study on PD patients and achieved an accuracy (±precision) of 2.8 (±2.4) cm/s [30].

From the lab assessment, gait speed was calculated for all walking tests (20-m walk test at normal and fast pace, and the circular walking test). Two strides at the beginning and end of the tests, respectively, were excluded to obtain steady-state gait speed values.

**Figure 2:**
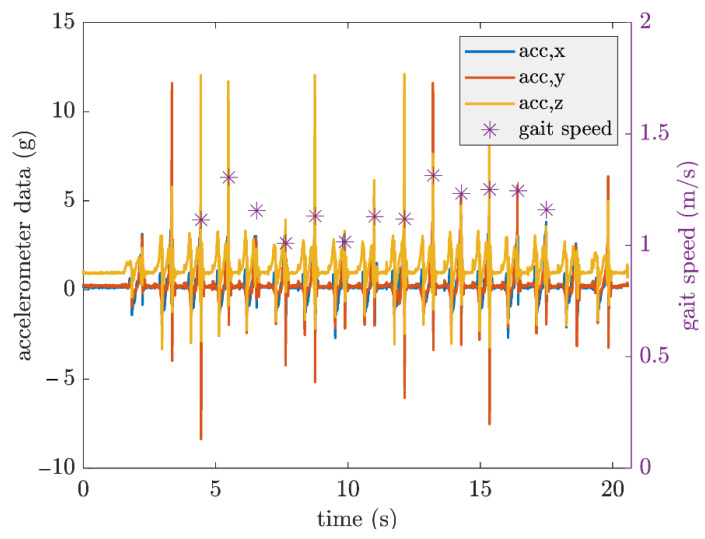
Example of gait speed extraction from a PD patient during straight walking at fast pace. Gait speed is given for each gait cycle of the right foot.

As described in the reference [29], the raw accelerometer and gyroscope data were processed to first detect the gait events. The acceleration of the movement was integrated to obtain the velocity of the foot. During the motionless periods, the zero-velocity update approach was applied to overcome the drift problem. Gait speed was then calculated from the drift-free velocity. Details of the procedure are provided in [29]. An example of extracted data is shown in Figure 2.

From the home assessment, due to the complex context of the unsupervised setting, and the vast distribution of gait speed [24], two approaches were employed. In the first approach, gait speed was obtained from each stride. In the second approach, mean gait speed per walking bout (WB) was calculated. WBs were determined as described earlier [31], and then divided into short (15–30 s), medium (> 30–60 s) and long WBs (>60 s). WBs shorter than 15 s were not included to avoid any influence on the accuracy of the used algorithm. Gait cycles having less than 0.2 m/s of gait speed were not considered, as they can be assumed to be static periods. Gait bouts were then allocated to respective ON and not-ON states: ON state was arbitrarily defined as 60–180 min after dopaminergic medication intake (based on the dopaminergic intake time in the diary). The period between 30 min before and 30 min after dopaminergic medication intake was defined as not-ON state, describing the condition in which no optimal drug effect is to be assumed [32,33,34].

### 2.4. Statistical Analysis

From the lab data, mean gait speeds of each trial were extracted. From home-based data, the 25th, 50th, 75th, 90th percentiles and maximum values of gait speeds were calculated from all gait cycles. The use of different percentiles was based on previous studies showing a heterogeneous distribution of daily-life gait speed in the elderly, resulting in relevant correlations with higher percentiles with capacity in the lab [35].

From the WB approach, we calculated the mean gait speed within each WB type, and the corresponding 25th, 50th and maximum values of each WBs type were considered for both ON and not-ON medication states [35].

Normality of data was checked with Shapiro–Wilk test. To compare different medication states in the lab (ON, OFF) and home environment (ON, not-ON), a paired comparison (t-test for parametric data; Wilcoxon signed rank test for non-parametric data) was used, and *p* values < 0.05 were considered significant.

In order to compare PD patients’ gait speed at home and in the lab at both medication states, respectively, and in relation to clinical scores, we performed a correlation analysis (Pearson correlation for parametric data; Spearman correlation for non-parametric data). A correlation coefficient of less than 0.5 was considered as low, between 0.5 and 0.7 as moderate and above 0.7 as high [36]. In addition, to measure the proportion of the variance of home assessment that is predictable from the lab assessments, the coefficient of determination (R^2^) was calculated, applying data transformation for non-normally distributed variables. All statistical analysis was performed using IBM^®^ SPSS 25 package.

## 3. Results

### 3.1. Demographic and Clinical Characteristics

A total of 39 PD patients were initially recruited. Out of this group, a total of 27 patients (40.7% female) met the inclusion criteria and performed the entire study protocol. Included participants did not significantly differ in demographic and clinical characteristics from those not included (Supplementary Table S1). Median age of the included participants was 69 years and the median disease duration was six years. Seventeen PD patients (63%) were early stage (Hoehn and Yahr stage ≤2 during medication OFF state), and 10 patients (37%) at mild-to-moderate stage (Hoehn and Yahrstage 2.5–3). Demographic and clinical details are provided in Table 1.

### 3.2. Gait Speed

#### 3.2.1. Comparison of Gait Speeds between Respective ON and OFF/not-ON States

**Figure 3:**
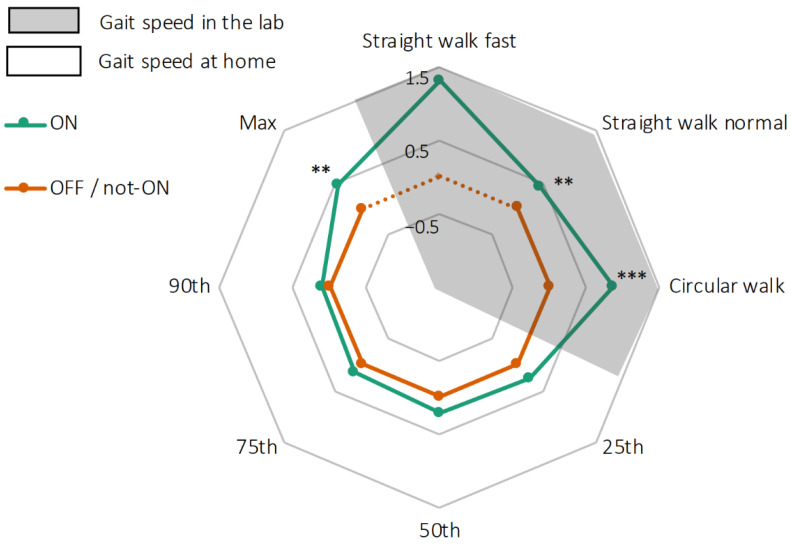
Radar plot illustrating gait speeds of 27 PD patients during lab tests (in grey) and in their domestic environment (white, presented by different percentiles). Gait speeds during medication OFF in the lab and not-ON in the domestic environment are presented as 0 (orange line), and gait speeds during medication ON are presented as deviation from 0 (green line). Straight walking test at fast pace was not performed during OFF medication state (dotted orange line). ** *p* < 0.01, *** *p* < 0.001.

Relative gait speeds in the lab and at home are shown in Figure 3. In the lab, straight walking at normal pace had a median value of 1.01 m/s during the medication ON state, and 0.97 m/s during the medication OFF state (*p* = 0.004). During circular walking it reached 0.69 m/s during the ON, and 0.58 m/s during the OFF state (*p* < 0.001). Only two PD patients were able to perform the straight walking at fast pace assessment during the medication OFF state; therefore, this task was not included in the analysis.

In the domestic environment, the median gait speed was 0.83 m/s during the medication ON state and 0.77 m/s during the medication not-ON state (*p* = 0.302). A significant difference was found between ON and not-ON medication states for the maximum gait speed (ON = 1.48 m/s; not-ON = 1.36 m/s; *p* = 0.009), but not for the other percentiles (Figure 3).

#### 3.2.2. Comparison of Gait Speeds between Lab and Home Environment during Medication ON State

During the medication ON states, low correlations were found when comparing gait speeds obtained from the normal walking tasks in the lab with the maximum values of home gait speed (*r* = 0.46; *p* = 0.02). Moderate correlations of gait speeds were observed between the fast walking task in the lab and the 90th percentile (*r* = 0.64; *p* < 0.001) and maximum values (*r* = 0.69; *p* < 0.001) of the home-derived data. Similar results were found when comparing the circular walking task in the lab with the 90th percentile (r = 0.53; *p* = 0.004) and maximum values (*r* = 0.61; *p* = 0.001). In general, the degrees of correlation between lab and home gait speeds increased with higher percentiles of gait speed in the unsupervised environment. This was also reflected by the R^2^ values (Table 2; Figure S1).

#### 3.2.3. Comparison of Gait Speeds between Lab and Home Environment during Medication OFF/not-ON State

During the medication OFF/not-ON states, moderate correlations were found when comparing gait speeds obtained from the normal walking tasks in the lab with the 25th percentile of home gait speed (*r* = 0.56; *p* = 0.004). Similar results were also found when comparing gait speed of the circular walking task in the lab with the 25th percentile of gait speed as obtained from the home data (*r* = 0.55; *p* = 0.004). This was also reflected by the R^2^ values (Table 2; Figure S1).

#### 3.2.4. Comparison of Gait Speeds between Lab and Home Environment during Opposite Medication States

Low correlations were found when comparing gait speeds obtained from the normal walking task in the lab at ON state with the maximum values of home gait speed at not-ON (*r* = 0.26; *p* = 0.209). Moderate correlations of gait speeds were observed between the fast walking task in the lab at ON and the maximum values of the home-derived data at not-ON (*r* = 0.47; *p* = 0.015). Similar results were found when comparing the circular walking task in the lab at ON with maximum value of home gait speed at not-ON (*r* = 0.41; *p* = 0.035) (Supplementary Table S2). The same lower percentiles were observed between measurements at OFF in the lab and ON in domestic environment: gait speed from the normal walking tasks at OFF and the 25th percentile from home at ON (*r* = 0.38; *p* = 0.063); circular walking task at OFF and the 25th percentile from home at ON (*r* = 0.43; *p* = 0.031).

### 3.3. Comparison of Gait Speeds in the Home Environment, Stratified by Different Bout Lengths

**Figure 4:**
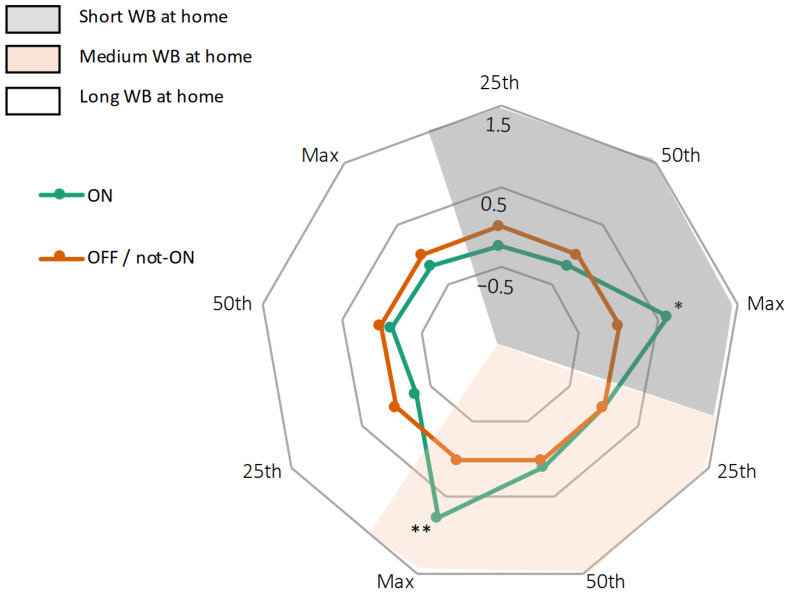
Radar plot illustrating gait speeds of 27 PD patients in their domestic environment, broken down into different WB. Gait speeds during medication not-ON are set at 0 (orange line), gait speeds during medication ON are presented as deviation from 0 (green line). * *p* < 0.05, ** *p* < 0.01.

In the home environment, WBs of different lengths most probably reflect different purposes of walking, such as doing the housework (short WBs) and taking a walk (long WBs) [37]. We found no significant differences in the number of WBs between ON and not-ON medication states (Supplementary Table S3). When comparing gait speeds between ON and not-ON states, stratified by different WB lengths, we found significant differences between ON and not-ON medication states only at high gait speeds. Moreover, this was only observed in short and medium WBs (short WBs: *p* = 0.026; medium WBs: *p* = 0.008). Long WBs did not add relevant information (Figure 4).

We then compared gait speeds from the lab with those obtained from the domestic environment during ON state, stratified by WB lengths. Degrees of correlation were highest between the fast walking task in the lab and the maximum gait speed during long WBs (*r* = 0.63; *p* < 0.001) and the circular walking task in the lab and the 50th percentile of gait speed during short WBs (*r* = 0.72; *p* < 0.001) (Table 3; Figure S2).

Consistently, when comparing gait speed between the lab and the domestic environment during OFF/not-ON state, stratified by WB lengths, degrees of correlation were highest between the normal walking task in the lab and the 25th percentile of gait speed during short WBs (*r* = 0.57; *p* = 0.004), and the circular walking task in the lab and the 25th percentile of gait speed during short WBs (*r* = 0.58; *p* = 0.003) (Table 3; Figure S2).

### 3.4. Comparison of Home-Collected Gait Speeds with UPDRS-III Scores

At ON state, there were no significant correlations between the UPDRS-III ON scores and the home-collected gait speed percentiles. Only item 30, assessing gait, significantly correlated with the 90th percentile of home-collected gait speed (*r* = −0.61; *p* = 0.001).

At not-ON state, there were no significant correlations between the UPDRS-III OFF scores and the home-collected gait speed percentiles at not-OFF. Only item 30 moderately correlated with the 25th percentile of home-collected gait speed (*r* = −0.44; *p* = 0.028).

## 4. Discussion

This exploratory cross-sectional study with PD patients during medication ON and OFF/not-ON states investigates gait speeds obtained from the lab and from a home assessment.

Firstly, gait speed was the only objective parameter considered in this analysis. Due to its combination of temporal and spatial gait characteristics, it is the most reliable predictor of mobility, and a valid and easy-to-administer measure of walking, which can reliably be estimated using IMUs [13].

Secondly, we decided to use different gait tasks in the lab and different set-ups in order to gather inertial signals similar to those that would be obtained in the daily life of PD patients. Other studies have only compared daily-life gait speed with tasks of short distances at normal pace, making this comparison less reliable [13,19,22,23]. We confirmed the importance of using both lab and home assessments to add considerable explanatory value to the understanding of PD motor function.

We found relevant differences in maximum gait speeds in the home environment between medication ON and not-ON states (Figure 1). When analyzing the best performance at home (i.e., the maximum values of gait speed at home) we were able to discriminate medication states. Moreover, when we analyzed gait speeds stratified by WB lengths, we found that maximum values of gait speed during short and medium WBs provided more informative to discriminate ON and not-ON states in PD. This finding suggests that maximum values of gait speed at home better represent the maximum capacity in the lab rather than normal daily-life performance. This may help clinicians to have a more precise estimation of the patient’s capacity when measuring mobility in unsupervised conditions. Considering extreme values of gait speed may be more informative when measuring mobility and the patient’s motor status. Furthermore, shorter walking bouts provide more discriminative information compared to longer walking bouts as short walking bouts might be accompanied by other cognitive or motor tasks. Therefore, we recommend considering these parameters as useful indicators when evaluating PD treatment’s effect in daily-life environments [22,24,37]. In unsupervised settings, the segmentation of WBs by length (short and medium WBs) provided additional information on individual patient ON/not-ON state.

We report, to the best of our knowledge, for the first time, that specific percentiles of gait speed and WB lengths may help in differentiating and monitoring ON and not-ON states in PD. Previous studies aimed to differentiate PD states in the home environment using other parameters rather than gait [38], or through the development of algorithms and machine learning approaches for quantifying specific PD motor symptoms, such as tremor, bradykinesia and dyskinesia [39,40,41,42]. Our study adds to such studies by including unsupervised gait speed performance as a relevant parameter to accurately monitor PD patients with mobile health technology, ultimately aiming at personalized adjustments in PD therapy.

We found that gait speed as assessed in medication ON state in the lab reflects (i) gait speeds obtained in the home during ON are better than during not-ON states, (ii) faster speeds correlated higher than slower speeds, and (iii) high correlations were mainly obtained in the long WBs. In more detail, we found the strongest association of fast pace in the lab with the highest gait speed percentiles (*r* = 0.69; *p* < 0.001) and long WBs (0.63; *p* = 0.002) of daily gait speed. This supports the concept that maximum capacity in the lab can efficiently reflect the best performance in daily life, and that assessments of capacity are possible in both lab and home environments.

In contrast, daily gait speed showed only low correlation with walking at preferred pace in the lab (*r* = 0.46). This lab gait task may thus not reliably reflect the complexity of mobility at ON state in everyday life, probably because asking to walk at ‘considered normal’ speed may lead to different interpretations, and cause a less homogeneous speed. Therefore, assessing fast speed in the lab may give more information on motor functions during daily-life activities. These discrepancies are also in line with previous studies: no significant correlation was found between walking at normal pace in the lab and home assessment [22], and lab-based gait assessment explained less than one third of the daily-living activity in PD [21]. This may be related to an increased awareness when performing tasks in a supervised environment or because of limited ecological validity when performing isolated movements [21,22,24]. Therefore, assessing fast speed in the lab may give more information on motor functions during daily-life activities.

We also observed that circular walking in the lab can moderately represent a patient’s everyday gait speed at home (*r* = 0.61). This association was pronounced in short WBs, which may reflect more complex and demanding gait situations in daily life, such as specific activities including walking and acting with the hands simultaneously [37,43]. Evidence from literature supports this hypothesis: gait features obtained in unsupervised conditions were closer to gait features obtained in the lab during dual-tasking, than when only walking [44,45,46,47].

In contrast, gait speed as assessed in medication OFF state in the lab reflects (i) gait speeds obtained in the home during not-ON are better than during ON states, (ii) slower speeds correlated higher than faster speeds, and (iii) high correlations were mainly obtained in the short WBs. In more detail, gait speed obtained during OFF state in the lab seems to best reflect how PD patients perform at home when below their usual performance, confirmed by the association of home-collected gait speed of lower percentiles (25th) and short WBs with all the lab tests (*r* = 0.56; *p* = 0.004). This is contrary to the results observed during respective ON states, where the higher was gait speed in the domestic environment, the higher was the correlation value with fast gait speed in the lab. Nevertheless, for this specific dataset, less information was obtained during not-ON state.

Taken together, these findings show that, during medication ON states, respectively, the fast speed gait value obtained in the lab can nicely inform about how PD patients perform their fast walking activities in their usual environment, and, similarly, circular walking can provide information about how PD patients behave in relation to more complex everyday tasks. By contrast, during medication OFF/not-ON states, the slow gait speed value as obtained in the lab can well inform about how PD patients perform their slow walking activities in their usual environment. We could also show a high discriminant validity of these findings because the respective opposite correlations did not show significant results (Supplementary Table S2).

These results are of clinical relevance as they suggest that straight walking at preferred speed in the lab may be substituted by alternative measurements if the aim is to collect information relevant for everyday life of PD patients at ON state. However, even if the most suitable assessment is performed, only about 40% of home gait speed could be explained by the lab assessment. This means that lab tests generally explain less than half of the home performance and, consequently, monitoring of daily-life gait seems to be of utmost relevance for a comprehensive understanding of PD gait in usual environments [21].

We also compared gait speed from real-life settings with the UPDRS measurements, which are based on a one-time physical examination. Only specific items of the UPDRS-III showed significant correlations with PD gait performance in everyday life. Comparably similar results were observed in a previous study [48], where no significant correlation was found among the entire UPDRS subgroups, but gait features showed significant correlations with specific items of the UPDRS-III, mostly related to gait. Therefore, it could be an advantage to assess PD mobility using objective and targeted parameters from lab and home-based tools.

The present study has some limitations. First, the sample size was relatively small. Second, unsupervised assessments were performed for only one day. We based this choice on the following considerations: a previous study [49] evaluated the repeatability of sensor-based assessments during two consecutive days and found that gait parameters were highly comparable between the two days. In addition, home and lab measures demonstrated strong discriminatory power in detecting impaired motor function in PD in another study, where unsupervised assessment was performed during one single day [22]. Still, further studies with several days of measurements are needed to capture a more granular picture of daily mobility [13]. Third, ON and not-ON states during daily-life activities were defined based on diary entries. Future studies should focus on a set-up that includes clearly defined OFF states (e.g., mornings, before medication intake).

We did not consider WBs shorter than 15 s, because we aimed to analyze steady-state gait and compare an equal number of steps with the lab capacity. However, in everyday situations, there are many shorter WBs occurring within the home or in-door conditions (<10 s) [11,37]. Since daily-living gait often takes place by using very short WBs, such bouts should also be considered in future analyses. Finally, the intra-subject variability of gait speed was considered only in the calculation of gait speed percentiles and not directly from the increment and decrement for each subject. Since it could be an interesting biomarker for the investigation of PD mobility, such analysis should be considered in further studies.

## 5. Conclusions

In-lab and daily-living testing with wearables can capture complementary aspects of PD, and substantially add to clinical evaluation and patient management. We highlighted which specific laboratory tests can better represent gait speed at home. This can be useful for clinicians, since it is possible to remotely assess the capacity of the patients in their domestic environment, and protect more vulnerable people, especially during the COVID-19 pandemic. On the other hand, if clinicians decide to perform only supervised tests, they know which functional tests are more indicative of patients’ daily-life performance. Another relevant highlight is the importance of including two different methods (percentiles of the total gait distribution and WB types) in the analysis of home-collected gait speed, for a more detailed representation of PD daily-life performance.

Improving the understanding of gait disabilities with additional information about daily-life performances from IMUs could enhance clinical care, design personalized interventions and overcome limitations concerning questionnaires and self-reported diaries.

## Figures and Tables

**Table 1:** Demographics and clinical characteristics of PD patients.

Variables	Value (IQR)
Male: Female	16:11
Age (years)	69 (64–73)
Disease duration (years)	6 (3–9)
Disease onset (years)	64 (57–69)
Hoehn and Yahr stage (0–5)	ON: 2 OFF: 2
UPDRS I (0–16)	2 (1–4)
UPDRS II (0–52)	7 (3–10)
UPDRS III (0–108)	ON: 12 (8–20) OFF: 22 (15–31)
UPDRS IV (0–23)	2 (0–3)
Total LED (mg)	580 (400–770)

Note: Results are expressed in median and interquartile range (IQR). LED: Levodopa equivalent dose; UPDRS: Unified Parkinson’s Disease Rating Scale.

**Table 2:** Correlation of ON and OFF/not-ON state between the lab and the domestic environment.

ON									
	Straight Walking Fast Pace			Straight Walking Normal Pace			Circular Walking		
	Comparison	Correlation	R^2^ (%)	Comparison	Correlation	R^2^ (%)	Comparison	Correlation	R^2^ (%)
	*p*-Value	*r*		*p*-Value	*r*		*p*-Value	*r*	
25th	<0.001	0.45 *	20	<0.001	0.38 *	14	0.715	0.36	13
50th	<0.001	0.54 **	29	0.003	0.40 *	16	<0.001	0.49 **	25
75th	<0.001	0.60 **	37	0.495	0.40 *	16	<0.001	0.53 **	28
90th	<0.001	0.64 ***	41	0.132	0.40 *	16	<0.001	0.53 **	28
Max	<0.001	0.69 ***	30	<0.001	0.46 *	15	<0.001	0.61 **	39
**OFF/not-ON**									
	**Straight Walking Fast Pace**			**Straight Walking Normal Pace**			**Circular Walking**		
	**Comparison**	**Correlation**	**R^2^ (%)**	**Comparison**	**Correlation**	**R^2^ (%)**	**Comparison**	**Correlation**	**R^2^ (%)**
	***p*-Value**	***r***		***p*-Value**	***r***		***p*-Value**	***r***	
25th	Straight walking test at fast pace was not performed during OFF medication state.			<0.001	0.56 **	33	0.038	0.55 **	31
50th				0.009	0.42 *	18	<0.001	0.39 *	16
75th				0.893	0.36	14	<0.001	0.38 *	15
90th				0.030	0.34	12	<0.001	0.41 *	17
Max				<0.001	0.15	2	<0.001	0.37	14

Paired comparisons (*p*-value), degrees of correlation (*r*) and coefficients of determination (R^2^) between lab tests and most relevant percentiles of gait speed in the domestic environment during ON and OFF/not-ON medication state. Correlation asterisks represent the following *p*-values: * *p* < 0.05, ** *p* < 0.01, *** *p* < 0.001.

**Table 3:** Correlation of ON and OFF/not-ON medication states between the lab and the domestic environment according to WB.

ON										
		Straight Walking Fast Pace			Straight Walking Normal Pace			Circular Walking		
		Comparison	Correlation	R^2^ (%)	Comparison	Correlation	R^2^ (%)	Comparison	Correlation	R^2^ (%)
		*p* Value	*r*		*p* Value	*r*		*p* Value	*r*	
Short	25th	<0.001	0.33 *	11	<0.001	0.31	10	<0.001	0.66 ***	44
	50th	<0.001	0.49 *	28	<0.001	0.32	10	<0.001	0.72 ***	52
	Max	<0.001	0.39 *	16	0.09	0.24	6	<0.001	0.49 **	24
Medium	25th	<0.001	0.17	3	<0.001	0.09	1	<0.001	0.51 **	26
	50th	<0.001	0.16	8	<0.001	0.05	0.3	0.532	0.39 *	16
	Max	<0.001	0.51 **	27	0.046	0.27	8	<0.001	0.40 *	17
Long	25th	<0.001	0.28	8	<0.001	0.24	6	0.04	0.45 *	21
	50th	<0.001	0.61 **	37	0.005	0.42*	18	<0.001	0.5 **	28
	Max	<0.001	0.63 **	41	0.649	0.29	9	<0.001	0.38 *	14
**OFF**										
		**Straight walking fast pace**			**Straight walking normal pace**			**Circular walking**		
		**Comparison**	**Correlation**	**R^2^ (%)**	**Comparison**	**Correlation**	**R^2^ (%)**	**Comparison**	**Correlation**	**R^2^ (%)**
		***p*-Value**	***r***		***p*-Value**	***r***		***p*-Value**	***r***	
Short	25th	Straight walking test at fast pace was not performed during OFF medication state.			<0.001	0.57 **	18	0.037	0.58 **	28
	50th				<0.001	0.39 *	16	0.617	0.44 **	19
	Max				0.02	0	0	<0.001	0.16	3
Medium	25th				<0.001	0.49 *	25	0.333	0.55 **	31
	50th				<0.001	0.51 *	27	0.006	0.50 *	26
	Max				<0.001	0.51 *	28	<0.001	0.55 *	28
Long	25th				0.028	0.38	15	<0.001	0.17	3
	50th				0.167	0.42	18	<0.001	0.18	4
	Max				0.322	0.2	4	<0.001	0.05	0

Paired comparisons (*p*-value), degrees of correlation (*r*) and coefficients of determination (R^2^) between lab tests and WB of gait speed in the domestic environment during ON and OFF/not-ON medication state. Correlation asterisks represent the following *p*-values: * *p* < 0.05, ** *p* < 0.01, *** *p* < 0.001.

## Data Availability

The data presented in this study are available on request from the corresponding author.

## Supplementary Materials

The following are available online at https://www.mdpi.com/article/10.3390/s21123974/s1, Figure S1: Correlation of ON and OFF/not-ON state between the lab and the most relevant percentiles of domestic environment, Figure S2: Correlation of ON and OFF/not-ON state between the lab and the most relevant WBs of domestic environment, Table S1: Demographic data of the patients included and excluded in the analysis, Table S2: Correlation of ON and OFF /not-ON states between the lab and the domestic environment, Table S3: Characteristics of WB during ON and OFF/not-ON medication states.
