# Supplementary material for: Comparison of Laboratory and Daily-Life Gait Speed Assessment during ON and OFF States in Parkinson’s Disease
Source: Sensors (Basel). 2021 Jun 9;21(12):3974. doi: 10.3390/s21123974 (PMC8229328; doi:10.3390/s21123974)
Supplement: Supplementary file 1 [file sensors-21-03974-s001.zip › sensors-1221217-supplementary.pdf]

Supplementary Materials:

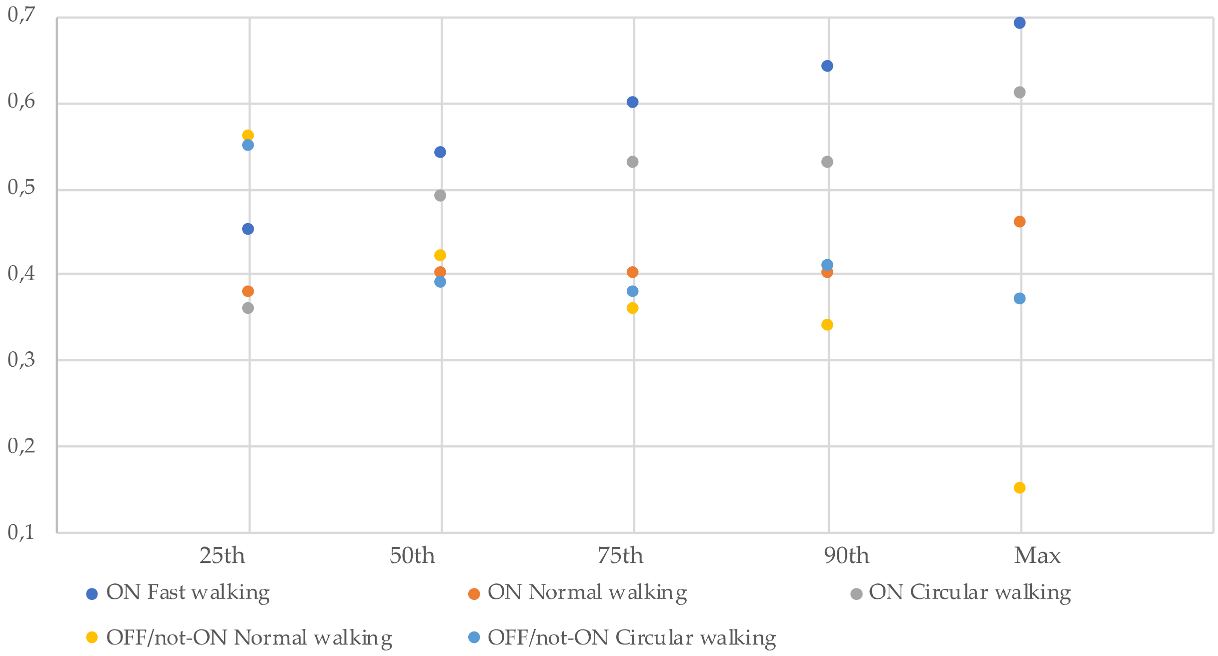

Figure S1. Correlation of ON and OFF/not-ON state between the lab and the most relevant percentiles of domestic environment.

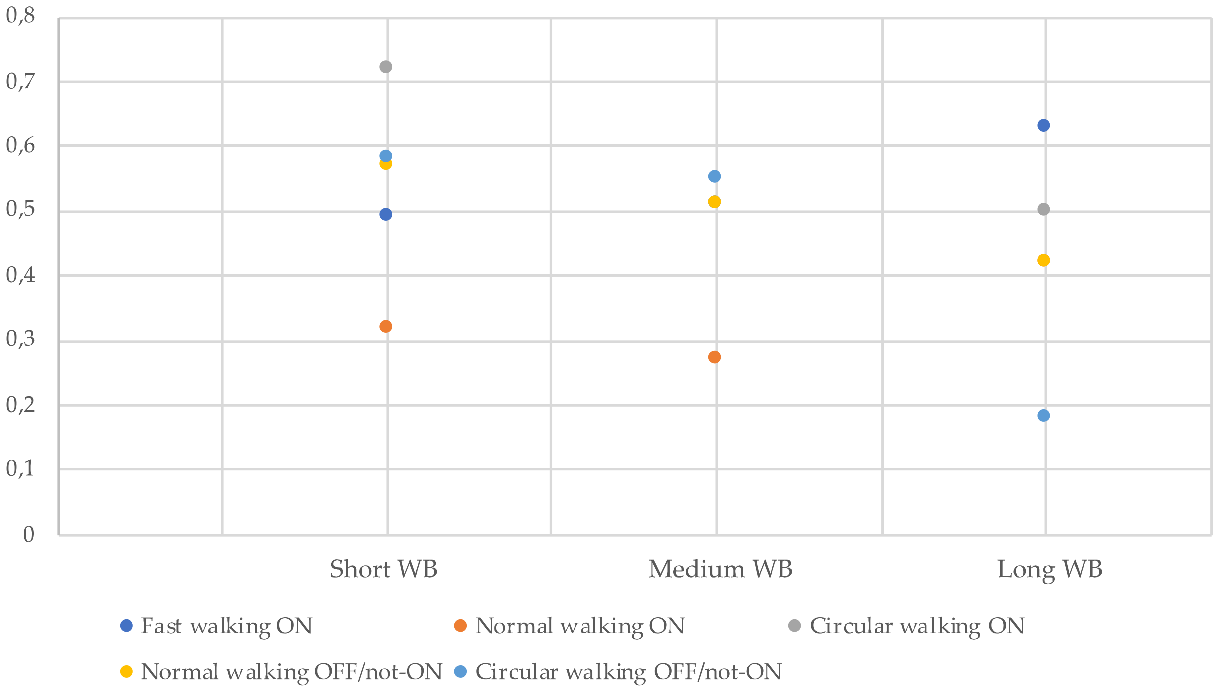

Figure S2. Correlation of ON and OFF/not-ON state between the lab and the most relevant WBs of domestic environment.

Table S1: Demographic data of the patients included and excluded in the analysis

**Variables**	**Included patients (27)**	**Excluded patients (12)**	***p* value**
Male:female	16:11	8:4	-
Age [years]	69 [64 : 73]	65 [61 : 74]	0.13
Disease duration [years]	6 [3 : 9]	4 [2 : 8]	0.3
Disease onset [years]	64 [57 : 69]	60 [53 : 69]	0.41
H&Y stage	**ON**: 2	2	0.39
	**OFF**: 2	2	0.06
UPDRS I	2 [1 : 4]	1 [1 : 2]	0.13
UPDRS II	7 [3 : 10]	4 [4 : 7]	0.23
UPDRS III	**ON**: 12 [8 : 20]	14 [8 :21]	0.5
	**OFF**: 22 [15 : 31]	22 [14 :33]	0.72
UPDRS IV	2 [0 : 3]	2 [0 : 3]	0.51
Total LED [mg]	580 [400 : 770]	540 [380 : 617]	0.36

Results are expressed in median and interquartile range [IQR].

Table S2: Correlation of ON and OFF / not-ON states between the lab and the domestic environment

**LAB**	Straight walking fast pace			Straight walking normal pace						Circular walking					
	ON			ON			OFF			ON			OFF		
**HOME**	P	r	R^2	P	r	R^2	P	r	R^2	P	r	R^2	P	r	R^2
ON	25th	0.45*	20	25th	0.38	14	25th	0.38*	14	25th	0.36	13	25th	0.43*	18
	Max	0.69***	30	Max	0.40*	16	Max	0.5*	13	Max	0.61**	39	Max	0.57**	27
Not-ON	25th	0.49*	24	25th	0.44*	19	25th	0.56**	33	25th	0.55**	30	25th	0.55**	31
	Max	0.47*	22	Max	0.26	7	Max	0.15	2	Max	0.41*	17	Max	0.37	14

Degrees of correlation (r) and coefficients of determination (R^2) between lab tests and most relevant percentiles (P) of gait speed in the domestic environment during ON and OFF / not-ON medication state. *p<0.05, **p<0.01, ***p<0.001.

Table S3: Characteristics of WB during ON and OFF / not-ON medication states.

	**ON**		**Not-ON**		***p value***
Time of ON and not-ON [hours]	5	[4 : 6]	2.5	[2 : 3]	<0.001
Walking time [%]	15.3	[10.4 : 26.3]	15.8	[9.3 : 18.7]	0.48
Total Short WB [N]	26	[11 : 42]	12	[7 : 23]	0.002
Short WB [N/h]	5.7	[3.4 : 7.5]	4.3	[2.8 : 9.4]	0.67
Total Medium WB [N]	9	[7 : 14]	5	[1 : 7]	<0.001
Medium WB [N/h]	2.3	[1.4 : 2,9]	1.7	[0.5 : 2.6]	0.27
Total Long WB [N]	5	[2 : 9]	2	[1 : 4]	0.024
Long WB [N/h]	1.3	[0.7 : 1.7]	0.7	[0.3 : 1.6]	0.51

Values are expressed in median [IQR].
